# Acral lentiginous melanoma in situ: dermoscopic features and management strategy

**DOI:** 10.1038/s41598-020-77425-z

**Published:** 2020-11-25

**Authors:** Byeol Han, Keunyoung Hur, Jungyoon Ohn, Sophie Soyeon Lim, Je-Ho Mun

**Affiliations:** 1 Department of Dermatology, Seoul National University College of Medicine, 101, Daehak-ro, Jongno-gu, Seoul, 03080 Republic of Korea; 2 Institute of Human-Environment Interface Biology, Seoul National University, Seoul, Republic of Korea; 3 School of Medicine, Monash University, Clayton, VIC Australia

**Keywords:** Cancer, Skin cancer, Skin diseases

## Abstract

Diagnosis of acral lentiginous melanoma in situ (ALMIS) is challenging. However, data regarding ALMIS are limited in the literature. The aim of this study was to investigate the clinical and dermoscopic features of ALMIS on palmoplantar surfaces. Patients with ALMIS and available dermoscopic images were retrospectively reviewed at our institution between January 2013 and February 2020. Clinical and dermoscopic features were analysed and compared between small (< 15 mm) and large (≥ 15 mm) ALMIS. Twenty-one patients with ALMIS were included in this study. Mean patient age was 58.5 (range 39–76) years; most lesions were located on the sole (90.5%). The mean maximal diameter was 19.9 ± 13.7 mm (mean ± standard deviation). Statistical analysis of dermoscopic features revealed that parallel ridge patterns (54.5% vs. 100%, P = 0.035), irregular diffuse pigmentation (27.3% vs. 100%, P = 0.001) and grey colour (18.2% vs. 90%, P = 0.002) were significantly less frequent in small lesions than in large lesions. We have also illustrated two unique cases of small ALMIS; their evolution and follow-up dermoscopic examination are provided. In conclusion, this study described detailed dermoscopic findings of ALMIS. Based on the present study and a review of the literature, we proposed a dermoscopic algorithm for the diagnosis of ALMIS.

## Introduction

Acral lentiginous melanoma (ALM), first described by Reed in 1976, is a histological subtype of cutaneous melanoma arising on the acral areas^1,2^. It is the most common type of melanoma in non-Caucasians; it is more aggressive and has a poorer prognosis than other subtypes of melanoma^3–7^. However, it is controversial whether ALM is biologically more aggressive than other subtypes; delayed diagnosis due to difficulty recognizing early ALM plays a major part in contributing to greater tumor thickness at diagnosis. Clinical diagnosis of ALM is challenging, especially in the early stages of ALM, where the clinical and histopathologic changes are subtle. Initial misdiagnosis and delay in diagnosis result in poorer patient outcomes; therefore, early detection and histopathologic correlation are important^8,9^.

Dermoscopy is a crucial tool for early detection of ALM. The presence of parallel ridge pattern (PRP) and irregular diffuse pigmentation (IDP) are characteristic dermoscopic features for ALM^10,11^. Previously, lesions that were clinically compatible with early ALM but lacked prominent histological atypical patterns were diagnosed as atypical melanosis of the foot and were deemed benign^9,12^. However, serial follow-up examinations and biopsies have revealed evolution of the lesion into acral lentiginous melanoma in situ (ALMIS)^9,13^. In addition, dermoscopic investigation revealed that PRP was observed in these lesions on initial presentation^13,14^. Therefore, atypical melanosis of the foot is now regarded as early ALMIS^9^. This historical conceptual change reflects difficulty diagnosing early stages of ALMIS due to the nonsubstantial morphologic changes and highlights the importance of detailed observation using new diagnostic tools. However, to our knowledge, there have been limited data specifically focusing on dermoscopic features of ALMIS on palmoplantar surfaces.

In this study, we investigated the clinical and dermoscopic findings of ALMIS cases at our institution. As clinical suspicion of ALM is routinely raised for large acquired acral melanocytic lesions, we instead focused on exploring the dermoscopic differences between small (< 15 mm) and large (≥ 15 mm) ALMIS. Lastly, we reported the evolution of two small ALMIS: a 4.5-mm and a 5-mm ALMIS from a 2.5-mm and a 3.5-mm pigmented macule detected after serial follow-up examination using photography and dermoscopy over 2 years and 1 year, respectively. These cases demonstrate the importance of close observation for acquired melanocytic lesions with non-typical dermoscopic features for the early detection of malignant lesions on glabrous skin.

## Results

Twenty-one patients with ALMIS were included in this study. The mean age was 58.5 (range 39–76) years, and 18 patients were women (85.7%). Most lesions were located on the sole (19 cases, 90.5%) and two lesions were on the palm (9.5%). Mean maximal lesion size was 19.9 ± 13.7 (mean ± SD) mm and mean duration of onset before diagnosis was 6.9 ± 7.4 (mean ± SD) years (Table 1).

**Table 1:** Demographics and dermoscopic features of acral lentiginous melanoma in situ with analysis between the small (< 15 mm) and large (≥ 15 mm) group.

Variable	Mean (SD) N = 21	Diameter		*P*
		< 15 mm N = 11	≥ 15 mm N = 10	
Age, years	58.5 (10.9)	51.9 (10.7)	65.8 (5.1)	**0.013**
Diameter, mm	19.9 (13.7)	9.4 (3.7)	31.4 (10.8)	** < 0.001**

	n (%)	n (%)	n (%)	
**Sex**				
Female	18 (85.7)	9 (81.8)	9 (90)	1.000
Male	3 (14.3)	2 (18.2)	1 (10)	
**Anatomic location**				
Sole	19 (90.5)	9 (81.8)	10 (100)	0.476
Palm	2 (9.5)	2 (18.2)	0	
**Asymmetry**	21 (100)	11 (100)	10 (100)	
1 axis	1 (4.8)	1 (9.1)	0	1.000
2 axes	20 (95.2)	10 (90.9)	10 (100)	1.000
**Number of colours**				
1	4 (19)	4 (36.4)	0	0.187
2	10 (47.6)	5 (45.5)	5 (50)	
3	5 (23.8)	2 (18.2)	3 (30)	
4	1 (4.8)	0	1 (10)	
5	1 (4.8)	0	1 (10)	
**Colour**				
Black	11 (52.4)	7 (63.6)	4 (40)	0.395
Brown	20 (95.2)	10 (90.9)	10 (100)	1.000
Grey	11 (52.4)	2 (18.2)	9 (90)	**0.002**
Blue	1 (4.8)	0	1 (10)	0.476
White	5 (23.8)	1 (9.1)	4 (40)	0.149
Red	0	0	0	–
**Pattern**				
Parallel ridge pattern	16 (76.2)	6 (54.5)	10 (100)	**0.035**
Irregular diffuse pigmentation	13 (61.9)	3 (27.3)	10 (100)	**0.001**
Irregular dots and globules	7 (33.3)	5 (45.5)	2 (20)	0.361
Irregular blotches	3 (14.3)	2 (18.2)	1 (10)	1.000
Irregular fibrillar pattern	6 (28.6)	3 (27.3)	3 (30)	1.000
Regression	2 (9.5)	1 (9.1)	1 (10)	1.000
Blue-white veils	1 (4.8)	0	1 (10)	0.476
Polychromia	1 (4.8)	0	1 (10)	0.476
Hyperkeratosis	3 (14.3)	0	3 (30)	0.090
Non-typical pattern	3 (14.3)	3 (27.3)	0	0.214

Bold font indicates statistical significance.

All lesions showed asymmetry; asymmetry was found in the majority of cases in two axes (20 cases, 95.2%) except in one case that had asymmetry in one axis (4.8%). More than two colours were found in 17 cases (81%). The combination of colours were two in ten (47.6%), three in five (23.8%), four in one (4.8%) and five in one case (4.8%). The most commonly detected colour in ALMIS was brown (95.2%), followed by grey (52.4%), black (52.4%), white (23.8%), and blue (4.8%). PRP was the most commonly detected dermoscopic pattern (76.2%). Other patterns were also observed, including IDP (61.9%), irregular dots and globules (33.3%), irregular fibrillar pattern (28.6%), irregular blotches (14.3%), hyperkeratosis (14.3%), regression (9.5%), blue-white veil (4.8%), and polychromia (4.8%). Ulceration or atypical vascular pattern was not found. Three cases (14.3%) showed a non-typical pattern.

**Figure 1:**
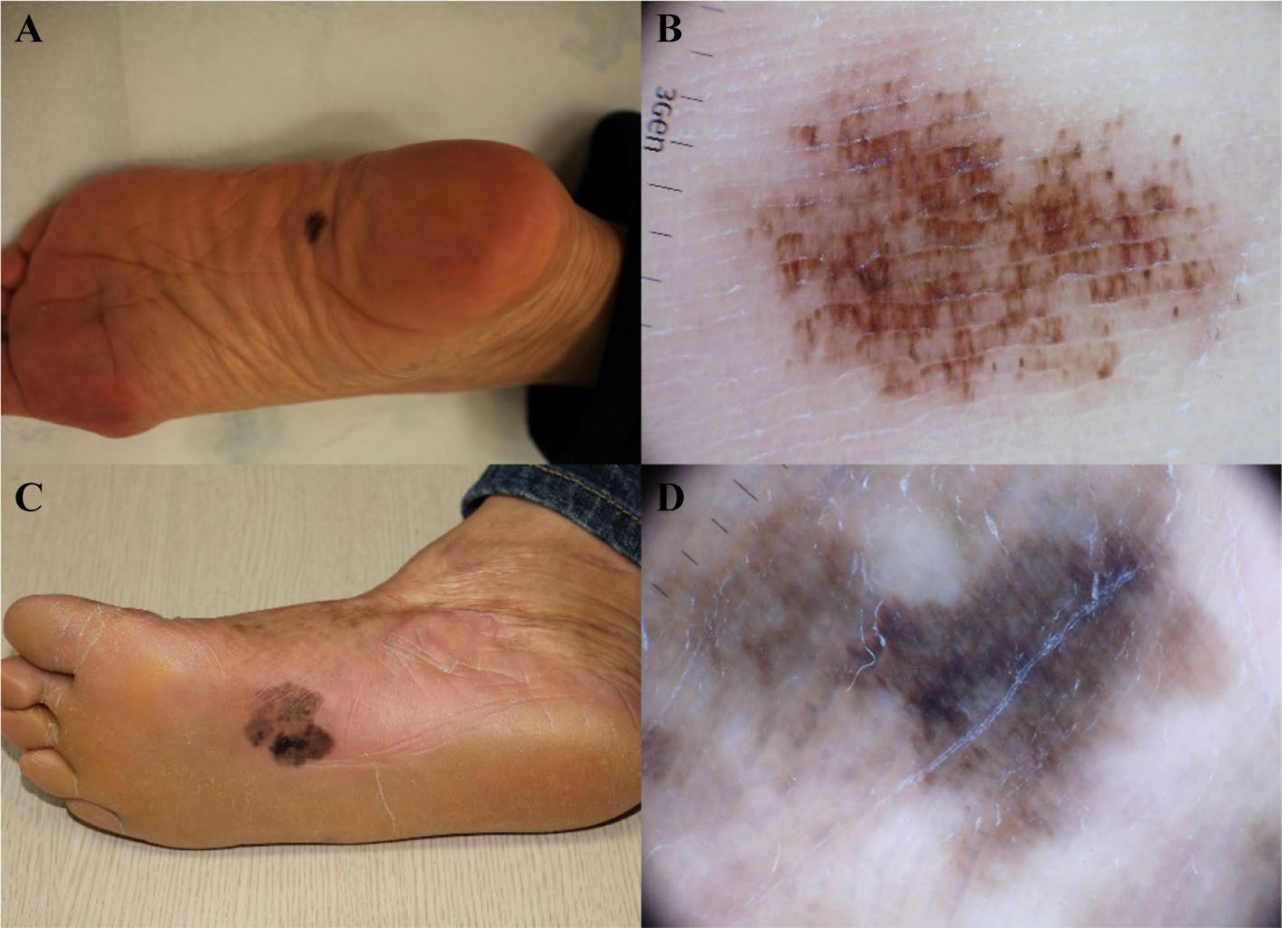
Clinical and dermoscopic features of acral lentiginous melanoma in situ (ALMIS): small versus large ALMIS. **(A,B)** The 12-mm ALMIS showed asymmetry, irregular dots and globules, and irregular fibrillar pattern. **(C,D)** The 31-mm ALMIS showed asymmetry, parallel ridge pattern, and irregular diffuse pigmentation.

When we divided the lesions according to their diameter, 11 cases (52.4%) were in the small group (diameter < 15 mm) and 10 cases (47.6%) were in the large group (diameter ≥ 15 mm) (Fig. 1). Patients in the small group (mean age 51.9 years) were younger than patients in the large group (mean age 65.8 years) (*P* = 0.013). Most cases in the small group (81.8%) had one or two colours, whereas there was greater variegation in the large group. Statistical analysis of dermoscopic features revealed that PRP (54.5% versus 100%, *P* = 0.035), IDP (27.3% vs. 100%, *P* = 0.001) and grey colour (18.2% vs. 90%, *P* = 0.002) were significantly less frequent in small lesions than in large lesions.

### Small ALMIS evolution detected during follow-up photography and dermoscopy

**Figure 2:**
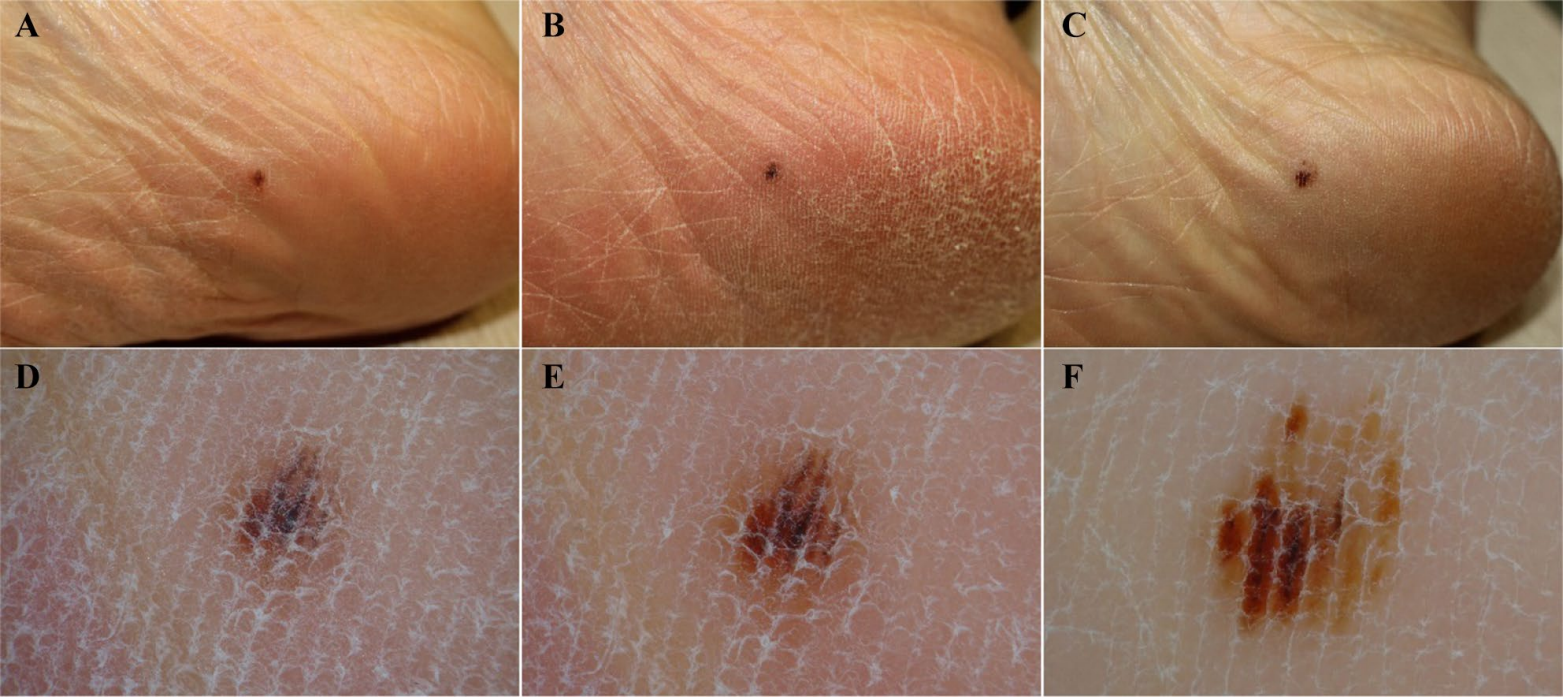
Serial clinical and dermoscopic findings of ALMIS on the sole in a 47-year-old woman over 24 months. **(A,D)** At the first visit, a 2.5-mm brown macule with non-typical pattern was seen. **(B,E)** After 6 months, the lesion increased to 3 mm with darkening of colours. **(C,F)** At the 24-month follow-up visit, the lesion enlarged to 4.5 mm and parallel ridge pattern and asymmetry were seen on dermoscopy.

Two small pigmented macules were diagnosed as ALMIS during their follow-up visit. The first case was seen in a 47-year-old woman. She presented with a 2-year history of a 2.5-mm brown macule on her right sole. Non-typical pattern was detected on initial dermoscopic examination with pigmentation on furrows and ridges. Given the non-typical pattern and its small size, the lesion was assigned for follow-up examination. Six months later, the lesion grew to 3 mm, and the background brown colour became clear. In the visit after 24 months, the lesion had grown to 4.5 mm and morphologic patterns had evolved with multicolour pigmentation (light brown, dark brown, and black), more marked asymmetry, and unequivocal PRP (Fig. 2).

Consequently, the lesion was excised due to suspicion of small ALMIS. Histopathologic analysis confirmed ALMIS, detecting atypical melanocytic proliferation in a single cell predominant pattern. The melanocytes were hyperchromatic and distributed in a lentiginous and non-cohesive nested pattern. Pagetoid melanocytes were stained positively with HMB-45 (Supplemental Fig. 1).

The second case was found in a 48-year-old woman who presented with a one-year history of a new 3.5-mm right sole lesion. Non-typical pattern was detected on dermoscopy. Follow-up examination was conducted at 6 and 12 months. Serial dermoscopic analysis showed growth in size (5 mm) and evolution of irregular fibrillar pattern with asymmetry. Eventually, the lesion was excised with 3-mm margins at the 12-month follow-up visit. Histopathologic findings were compatible with a diagnosis of ALMIS.

## Discussion

Dermoscopy has revolutionised clinical diagnosis of ALM by enabling detailed morphologic analysis of pigmented patterns. In this study, we found that grey colour, PRP, and IDP were significantly less common in small ALMIS than in large ALMIS (*P* = 0.002, *P* = 0.035, and *P* = 0.001, respectively).

PRP is the cardinal dermoscopic feature of ALM^9,10,17,19^. It is defined as prominent band-like pigmentation on the ridges of the dermatoglyphic lines^10^. Although it is the most well-known important dermoscopic features of ALM, PRP was absent in four cases of the small group (36.4%) in our data. Upon further analysis of small ALMIS without PRP, they showed other malignant features: irregular dots and globules in three, irregular fibrillar pattern in two, and IDP in one. Therefore, careful investigation of melanoma-specific dermoscopic patterns in addition to PRP is important especially in small lesions. IDP with variable shades from tan and grey to black colour is also specific to ALM^10,17^. Thirteen cases (61.9%) had IDP. This pattern was more prevalent in large lesions.

Early detection is crucial in improving melanoma prognosis. Saida and Koga proposed that a cut-off of larger than 7 mm is a helpful clue for ALM and incorporated this criterion in the three-step algorithm for early ALM screening^20^. Nonetheless, as ALMs generally arise de novo^9,21^, recognising small evolving lesions is essential for adequate management. However, the current literature on this topic is scarce. Data from Mayo clinic suggested that 6-mm acral melanomas could be missed when using the three-step algorithm^22^. Moreover, we reported two exceptional cases of evolving small ALMIS (4.5 mm and 5 mm). The cases were initially difficult to diagnose because they showed non-typical patterns. However, follow-up dermoscopic examination revealed increase in size and changing dermoscopic patterns. These cases highlight the importance of detecting small evolving pigmented macules for optimising ALM management.

**Figure 3:**
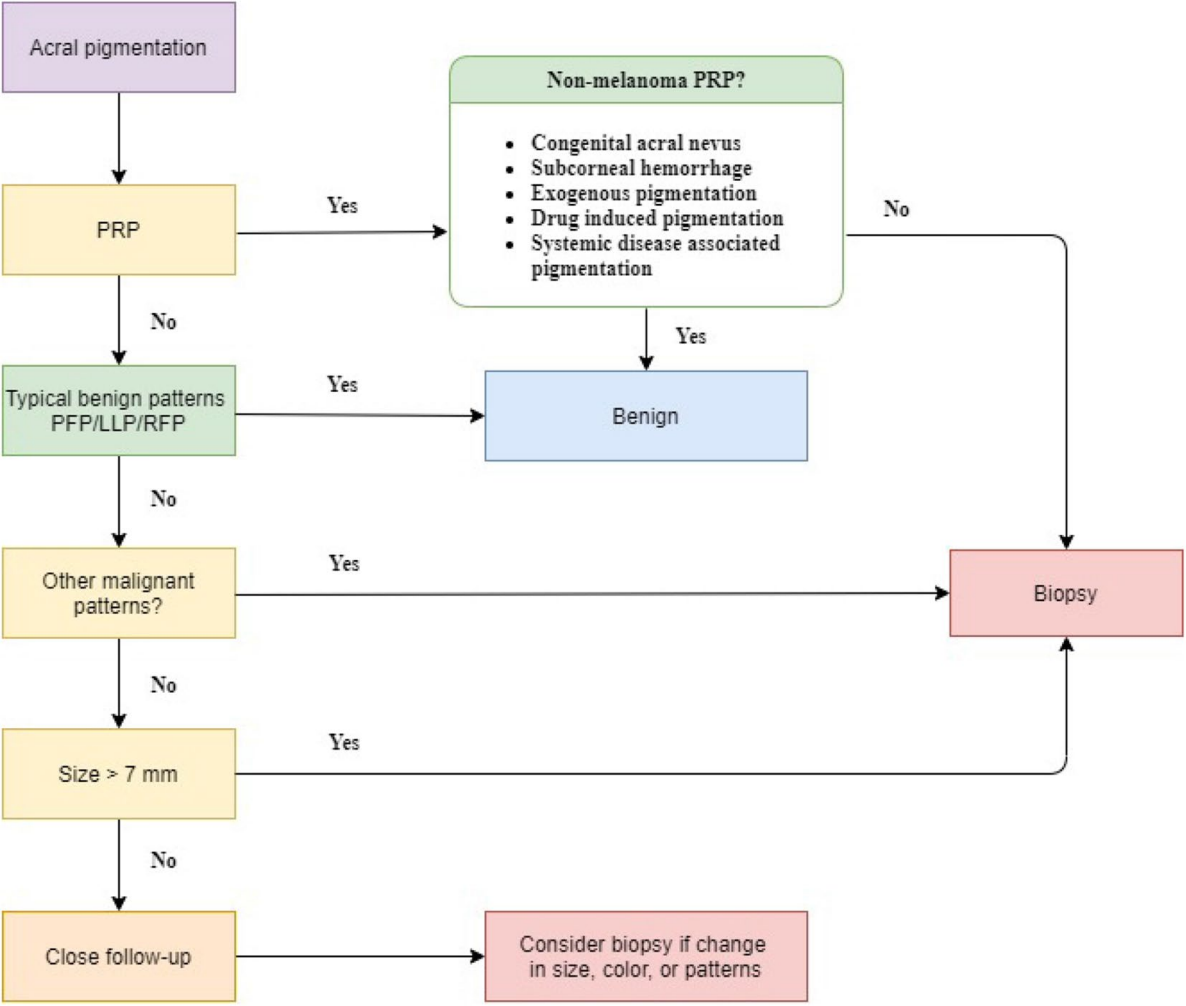
Our proposed management algorithm for acral lentiginous melanoma in situ. Abbreviations: LLP, lattice-like pattern; PFP, parallel furrow pattern; PRP, parallel ridge pattern; RFP, Regular fibrillar pattern.

Based on our data and the literature, we suggest the following steps for management of acral pigmented lesions (Fig. 3). When a patient presents with a melanocytic lesion on glabrous skin, the clinician should first evaluate for PRP. If PRP is present, the clinician must rule out benign diseases showing PRP such as acral subcorneal haemorrhage, exogenous pigmentation including para-phenylenediamine, chemotherapy-induced hyperpigmentation, hereditary syndromes such as Peutz-Jeghers syndrome or Laugier-Hunziker syndrome, acral Spitz nevus, and congenital melanocytic nevus^23–26^. If there is no evidence of benign causes of PRP, prompt biopsy is warranted.

In a melanocytic lesion on the glabrous skin without PRP, the presence of typical benign patterns (parallel furrow, lattice-like, regular fibrillar) should be evaluated. If these patterns are present, the lesion is benign and no further treatment is necessary. If not, this lesion is described as having a non-typical pattern. The clinician should then evaluate for other malignant features. If there is asymmetry, multicolour, IDP, irregular dots and globules, blotches, irregular fibrillar pattern, regression, blue-white veil, or atypical vascular patterns, clinicians should consider histopathologic examination. If these malignant features are absent, clinicians should assess the lesion size. Lesions with > 7 mm diameter should be excised for histopathologic examination, while lesions with ≤ 7 mm diameter should be closely monitored. If the lesion shows any change in size, colour or pattern, biopsy should be considered.

This study has some limitations. First, the sample size is small. However, as ALMIS is rare, our data provides valuable insight into detailed dermoscopic patterns of ALMIS and their management. Further studies with a larger cohort are necessary. Second, selection bias may exist as cases with obvious dermoscopic features are easier to diagnose accurately.

In summary, we have presented the in-depth dermoscopic features of ALMIS. Grey colour, PRP, and IDP were significantly less frequent in small ALMIS. Additionally, we reported cases demonstrating evolution of small ALMIS. Therefore, careful attention is needed for new-onset melanocytic lesions on soles and palms in adults. Finally, we propose a dermoscopic algorithm for ALM based on the previous literature and our data.

## Methods

A retrospective, chart review, single-centre study was performed to investigate cases of ALMIS at Seoul National University Hospital, Seoul, Korea. This study was approved by the institutional review board of the Seoul National University Hospital (H-2003-168-1112). All research was performed in accordance with the Declaration of Helsinki. Cases with ALMIS on the soles and palms with available high-quality clinical and dermoscopic photography from January 2013 to February 2020 were included. All cases were diagnosed base on the our previously reported clinical, dermoscopic, and histopathologic criteria^9^. Nail apparatus melanomas were excluded as they have distinctive dermoscopic patterns. Dermoscopic photographs were taken with a DermLite II Pro HR, DL3, or DL4 equipment (3Gen, San Juan Capistrano, CA) coupled to a digital camera. Informed consent was obtained from all patients prior to biopsy.

Patient demographics and dermoscopic features of the lesions were evaluated. Colours (black, brown, grey, blue, red, and white) and ALM-associated patterns were investigated according to the previous literature, including PRP, IDP, asymmetry, irregular blotches, blue-white veil, irregular dots and globules, ulcers, regression structures, polychromia (≥ 4 colours), atypical vascular patterns, irregular fibrillar patterns, and hyperkeratosis^15–17^. Non-typical pattern was defined as absence of a parallel furrow pattern, lattice-like pattern, fibrillar pattern, and PRP^18^. Dermoscopic patterns were evaluated by two board certified dermatologists experienced in dermoscopy. Any disagreement was resolved in a consensus meeting. Cases were divided into two categories (small < 15 mm; large ≥ 15 mm) to analyse the dermoscopic differences depending on size (maximal diameter). Most cases were diagnosed upon initial presentation; however, two small cases were diagnosed upon follow-up dermoscopic examination. These unique cases of small ALMIS will be further discussed.

Descriptive statistics were used to evaluate the dermoscopic features of ALMIS. Pearson’s Chi-Square test or Fisher’s exact test (for < 5 cells expected in the software) was performed to compare proportions. The Mann–Whitney U test was used for continuous variables. All analyses were performed using SPSS 25 (SPSS Inc., IL, USA).

## Supplementary information

Supplementary Figure 1.
