# Supplementary material for: Acral lentiginous melanoma in situ: dermoscopic features and management strategy
Source: Sci Rep. 2020 Nov 25;10:20503. doi: 10.1038/s41598-020-77425-z (PMC7688656; doi:10.1038/s41598-020-77425-z)
Supplement: Supplementary file 1 — Supplementary Figure 1. [file 41598_2020_77425_MOESM1_ESM.docx]

Original research

Acral lentiginous melanoma in situ: Dermoscopic features and management strategy

Byeol Han, MD,^1,2^ Keunyoung Hur, MD,^1^ Jungyoon Ohn, MD,^1,2^ Sophie Soyeon Lim,^3^ Je-Ho Mun, MD, PhD^1,2*^

^1^Department of Dermatology, Seoul National University College of Medicine, Seoul, Republic of Korea

^2^Institute of Human-Environment Interface Biology, Seoul National University, Seoul, Republic of Korea

^3^School of Medicine, Monash University, Clayton, VIC, Australia

**
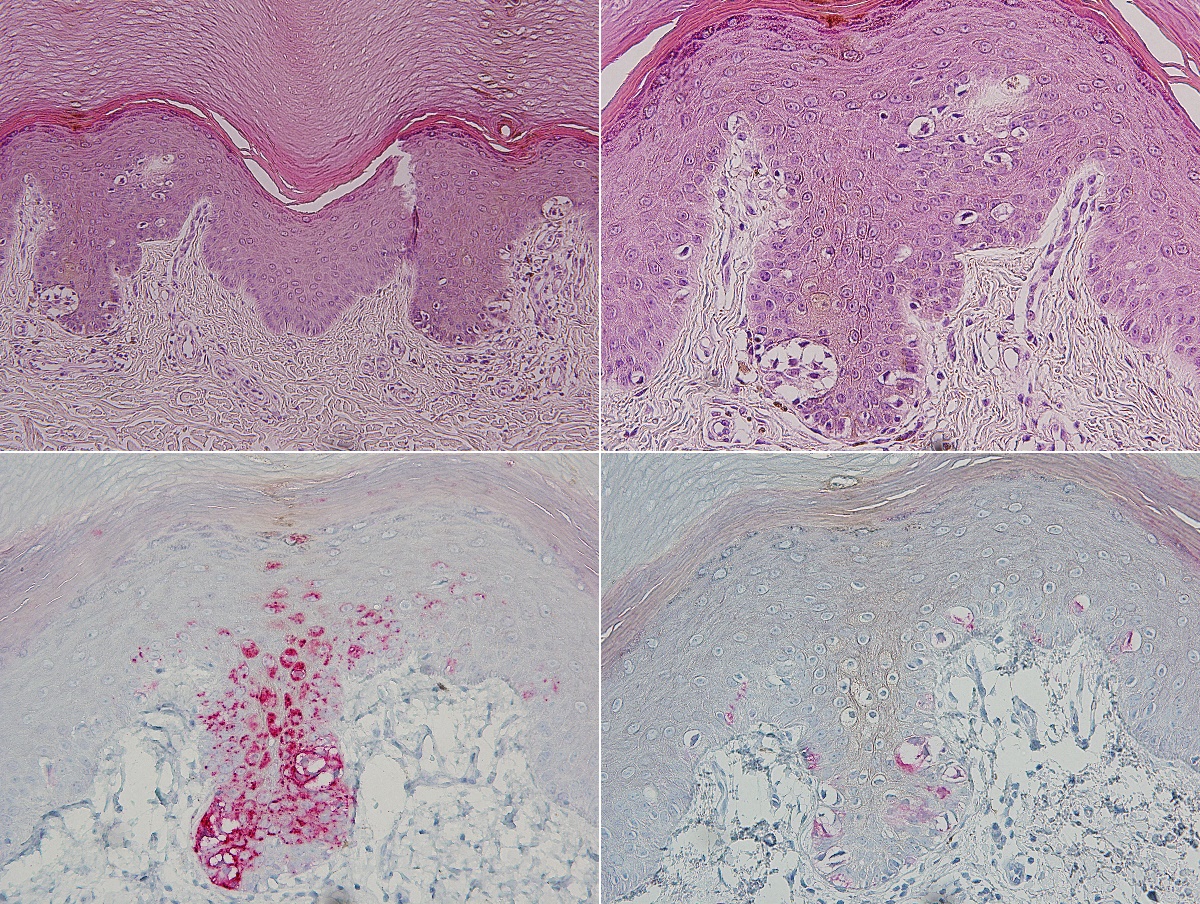
**

**Supplemental Fig. 1.** Histopathologic features of a 4.5-mm small ALMIS (A, B). Atypical melanocytes were scattered in a single cell predominant pattern with pagetoid spreading and non-cohesive nest formation (haematoxylin-eosin staining ×200 and ×400). (C, D) Atypical melanocytes were stained with HMB-45 (C, ×400) and less weakly with Melan-A (D, ×400).
